# Retraction: *In vivo* and *in vitro* effects of microRNA-27a on proliferation, migration and invasion of breast cancer cells through targeting of *SFRP1* gene via Wnt/β-catenin signaling pathway

**DOI:** 10.18632/oncotarget.28616

**Published:** 2024-08-14

**Authors:** Ling-Yu Kong, Mei Xue, Qing-Cai Zhang, Chuan-Fu Su

**Affiliations:** ^1^Department of Breast, Linyi Cancer Hospital, Linyi 276000, P.R. China; ^2^Department of Pathology, Linyi Cancer Hospital, Linyi 276000, P.R. China; ^3^Operating Theatre, Daqing Oilfield General Hospital, Daqing 163000, P.R. China

**This article has been retracted:** In Figure 7C, the second and sixth panels contain images duplicated from Figure 7 of [1]. This same figure also contains a partially duplicated panel from Figure 7C of a second paper, which has been retracted [2]. The journal requested clarification regarding these images from the authors. The authors have acknowledged that some images provided by a third-party lab may be unreliable, and thus the data is compromised. In light of these facts, the Editorial decision was made to retract this paper. All authors agreed with the decision. Linyi cancer hospital acknowledged the retraction of this paper.

Original article: Oncotarget. 2017; 8:15507–15519. 15507-15519. https://doi.org/10.18632/oncotarget.14662
